# DiapHRaGM: A mnemonic to describe the work of breathing in patients with respiratory failure

**DOI:** 10.1371/journal.pone.0179641

**Published:** 2017-07-03

**Authors:** Aiman Tulaimat, William E. Trick

**Affiliations:** 1 Division of Pulmonary, Critical Care, and Sleep Medicine, Department of Medicine, Cook County Health and Hospitals System, Chicago, Illinois, United States of America; 2 Collaborative Research Unit, Department of Medicine, Cook County Health and Hospitals System, Chicago, Illinois, United States of America; University of Bari, ITALY

## Abstract

**Background:**

The assessment of the work of breathing in the definitions of respiratory failure is vague and variable.

**Objective:**

Identify a parsimonious set of signs to describe the work of breathing in hypoxemic, acutely ill patients.

**Methods:**

We examined consecutive medical ICU patients receiving oxygen with a mask, non-invasive ventilation, or T-piece. A physician inspected each patient for 10 seconds, rated the level of respiratory distress, and then examined the patient for vital signs and 17 other physical signs. We used the rating of distress as a surrogate for measuring the work of breathing, constructed three multivariate models to identify the one with the smallest number of signs and largest explained variance, and validated it with bootstrap analysis.

**Results:**

We performed 402 observations on 240 patients. Respiratory distress was absent in 78, mild in 157, moderate in 107, and severe in 60. Respiratory rate, hypoxia, heart rate, and frequency of most signs increased as distress increased. Respiratory rate and hypoxia explained 43% of the variance in respiratory distress. Diaphoresis, gasping, and contraction of the sternomastoid explained an additional 28%. Heart rate, blood pressure, alertness, agitation, body posture, nasal flaring, audible breathing, cyanosis, tracheal tug, retractions, paradox, scalene or abdominal muscles contraction did not increase the explained variance in respiratory distress.

**Conclusion:**

Most of the variance is respiratory distress can be explained by five signs summarized by the mnemonic DiapHRaGM (*diap*horesis, *h*ypoxia, respiratory *ra*te, *g*asping, accessory *m*uscle). This set of signs may allow for efficient, standardized assessments of the work of breathing of hypoxic patients.

## Introduction

Tachypnea, abnormal blood gases, and increased work of breathing are the main manifestations of acute respiratory failure.[1] Respiratory rate and blood gases are easily and reliably measured and are interpreted according to physiologic principles.[2,3] In contrast, assessing the work of breathing is challenging. It cannot be easily measured at the bedside and physicians have to rely on their examination of the signs of increased breathing effort or on their gestalt of a patient’s effort—commonly referred to as respiratory distress.[2,4]

The definitions of respiratory failure in recent randomized trials demonstrate this challenge. The authors define the increased work of breathing with vague, variable terms: “labored breathing or respiratory distress or dyspnea at rest”,[5] “signs of high respiratory-muscle workload”,[6] and “signs suggestive of intense respiratory muscle work and\or labored breathing such as, use of accessory respiratory muscles, paradoxical motion of the abdomen, or intercostal retraction”.[7] These definitions assume that dyspnea, respiratory distress, and signs of increased breathing effort are interchangeable. They also disregard the number of signs present and divide patients into two mutually exclusive categories. These descriptions and similarly vague ones used in clinical practice underscore contemporary shortcomings of standardized measurement in clinical research and of communication in clinical practice.

Considering the consequences of respiratory failure, it is concerning that the clinical assessment of one of its critical features remains vague and challenging. Standardizing this assessment is desirable in clinical practice as well as research because it will potentially improve the quality of the physical examination of such patients and enhance the communication between physicians regarding its extent. To this end, our study aimed to identify the smallest number of physical signs that best describe the work of breathing in acutely ill hypoxic patients.

## Materials and methods

### Setting

The study was conducted at a 22-bed medical intensive care unit of a 450-bed teaching hospital. It was approved by the Institutional Review Board at John H. Stroger Hospital of Cook County (No. 06–159) and was conducted in accordance with the amended Declaration of Helsinki. Written consent was waived and a written notification about the study and the option to opt out were given to patients and families.

### Patients

We screened all patients daily to identify those receiving oxygen therapy by nasal cannula or mask, non-invasive ventilation, or undergoing a T-piece breathing trial to wean from mechanical ventilation. Patients were included more than once if they were still on the aforementioned respiratory support. We excluded patients receiving invasive mechanical ventilation. We extracted the diagnoses, demographics, and outcomes from the medical record.

### Physicians

We trained ten physicians on the steps of examining each patient and the method of detecting the signs (S1 Table). Five physicians were board certified in critical care medicine and five were critical care medicine fellows in their fifth or sixth year of training.

### Procedure

The heart rate (HR), systolic and diastolic blood pressures (SBP, DBP), and oxygen saturation by pulse oximetry (SpO_2_) were recorded from the monitor screen in the patient’s room before turning it off. A physician not involved in the patient’s care entered the private, well-lit room, looked at the patient for 10 seconds, and rated the level of respiratory distress into one of four levels (none, mild, moderate, or severe). The physician then examined each patient for 17 physical signs in a specified sequence (Table 1). Each respiratory sign was examined over five breath cycles. Their presence was categorized as definitely present, possibly present, possibly absent, or definitely absent.[8,9] Lastly, the physicians counted the respiratory rate (RR) over one or two minutes and recorded the fraction of inhaled oxygen (FIO_2_).[8,10]

**Table 1:** Sequence of physical examination.

Body posture indicating dyspnea
Audible breathing
Diaphoresis
Nasal flaring
Cyanosis
Gasping
Pursed lip breathing
Contraction of the scalene muscle
Contraction of the sternomastoid muscle
Tracheal tug
Retraction of the supraclavicular fossa
Retraction of the suprasternal fossa
Retraction of the lower ribs during inspiration (Hoover’s Sign)
Thoraco-abdominal asynchrony or paradox (referred to as paradox)
Contraction of abdominal muscles
Level of consciousness (alert, somnolent, stuporous, or comatose)
Agitation (calm, restless, agitated, or very agitated),
Respiratory rate (RR) counted over one or two minutes
Fraction of inhaled oxygen (FIO_2_)

### Analysis

This study was performed on a convenience sample of consecutive patients. We decided a priori to collect a minimum of 400 observations with a minimum of 60 observations in each of the four levels of distress. We did not perform a formal sample size calculation.

For most of the analyses, we re-classified the levels of respiratory distress by collapsing “no” and “mild” respiratory distress into a single category because the primary concern is to identify patients who have moderate or severe respiratory distress. To reduce uncertainty in interpreting a sign as “possibly” present or absent, we dichotomized the categories to definitely present versus all other categories.[9] We created a dummy variable to describe *hypoxia* (FIO_2_> 0.4 with SpO_2_<93%).

We evaluated the association between RR and the level of respiratory distress by constructing a lowess smoothed plots. We visually identified a RR > 24 breaths/min as a departure from linearity above which the likelihood of respiratory distress increased. Because increments of 5 breaths/min would be easy to recall and because it satisfied the proportional odds assumption for ordinal outcomes, we created five categories of RR (i.e., ≤24, 25 to 29, 30 to 34, 35 to 39, ≥40). We also created dichotomous “dummy” variables for HR (> 130 beats/min vs. ≤130 beats/min), level of *agitation* (calm vs. [restless, agitated, or very agitated]), and level of consciousness (alert vs. [somnolent, stuporous, or comatose]).[11]

We assessed the bivariable association of the level of respiratory distress with the signs and physiologic measurements by describing the proportion of patients in each level of respiratory distress (none or mild, moderate, or severe) for each measurement or sign. All variables significantly associated with respiratory distress using a non-parametric test for trend across the levels of respiratory distress were evaluated in multiple regression models.[12] The dependent variable was the 3-category level of respiratory distress.

We constructed three separate models that varied in the complexity of obtaining data: a physiologic model (vital signs and hypoxia); a limited model (physiologic model plus readily observable signs); and a full model (physiologic model plus all signs). Although heart rate, hypoxia, and respiratory rate are not signs of breathing effort, we tested them in the physiologic model to determine if they contributed in describing the level of distress. Readily observable signs included all of the signs except Hoover’s sign, paradox, and abdominal muscle contraction. Detecting them required the physician to remove a patient’s gown or change a patient’s position to palpate the chest or abdomen or both.

To account for the correlated data structure of our dataset—patients could be enrolled more than once—and to allow for the ordinal structure of our outcome, we used ordinal regression and specified the patient as a repeat observation in the variance-covariance matrix.[13] We compared the three models by calculating the McKelvey and Zavoina R-square value for estimates of model fit. Since it was our goal to build a parsimonious final model, i.e., the model with a minimum number of independent variables; we evaluated the model fit after iteratively removing each variable.[14] After establishing a final model, we subsequently evaluated the model fit after re-entering each variable into this final model. To evaluate the internal validity of our model we obtained parameter estimates using the bootstrap procedure and specified 500 replications of the dataset. Analyses was performed using version 10.1 of Stata (Stata, Inc., College Station, TX.). Raw physical examination data is available in the supporting information for this manuscript (S1 Data).

## Results

### Patients

We performed 402 observations on 240 patients: 260 by attending physicians and 142 by fellows. Most (89%) observations occurred in patients breathing spontaneously, 7% were during weaning, and 4% in patients breathing with non-invasive ventilation. Forty-five observations occurred at the time of intubation for mechanical ventilation.

The patients were 50±15 years old and 60% were men. The patients had various disorders of which 46% were respiratory (Table 2). The length of hospital stay was 14±12 days, 16% died in the intensive care unit, and 21% died during the hospitalization. Most patients were tachypneic and tachycardic and on moderate to high levels of oxygen support: RR, 28±10 breaths/min; HR, 98±20 beats/min; FIO_2_ 0.47 ±0.26.

**Table 2:** Primary diagnosis.

Diagnosis	n	%
Pneumonia	61	25.4
Advanced thoracic malignancy	22	9.2
Metabolic encephalopathy	22	9.2
Obstructive lung disease	21	8.8
Septic shock	17	7.1
Stroke (ischemic or hemorrhagic)	12	5.0
Acute respiratory distress syndrome	10	4.2
Hypercapnic respiratory failure	9	3.8
Cardiogenic pulmonary edema	8	3.3
Gastrointestinal bleeding	8	3.3
Complications of liver disease	7	2.9
Severe pancreatitis	7	2.9
Others	37	15.5

### Physiologic variables and physical examination

There was a significant monotonic increase in both the HR and RR across the levels of respiratory distress (Table 3). Hypoxia correlated strongly with the level of respiratory distress (Table 4). The blood pressure was similar across the levels of distress.

**Table 3:** Vital signs and the level of respiratory distress.

Respiratory Distress	RR	HR	SBP	DBP
**None (n = 78)**	21±6	87±18	129±23	72±13
**Mild (n = 157)**	25±6	95±17	131±25	73±14
**Moderate (n = 107)**	32±9	104±17	132±28	73±15
**Severe (n = 60)**	39±9	112±22	134±27	75±13

RR = respiratory rate in breaths/minute. HR = heart rate in beats per minute. SBP and DBP = systolic and diastolic pressures in mmHg. All the values are presented as mean±SD.

**Table 4:** Distribution of physiologic and physical signs present by level of respiratory distress.

	Category of Respiratory Distress					
	None or mild (n = 235)		Moderate (n = 107)		Severe (n = 60)	
	n	%	n	%	n	%
**Physiologic variables**						
Hypoxia^c^	5	2	10	9	18	30
Heart rate > 130 bpm^c^	2	1	9	8	15	25
Respiratory rate						
RR ≤ 24	134	57	22	21	3	5
RR 25 to 29	50	21	17	16	4	7
RR 30 to 34	36	15	28	26	9	15
RR 35 to 39	12	5	26	24	16	27
RR ≥ 40	3	1	14	13	28	47
**Respiratory signs**						
Pursed lips^b^	4	2	5	5	5	8
Gasping^c^	8	3	28	26	39	65
Posture indicating dyspnea^c^	11	5	9	8	23	38
Hoover’s sign^a^	11	5	16	15	5	8
Sternomastoid contraction^c^	21	9	37	35	48	80
Abdominal muscle contraction^c^	24	10	26	24	35	58
Audible breathing^c^	33	14	38	36	29	48
Paradox^c^	33	14	38	36	45	75
Tracheal tug^c^	36	15	49	46	48	80
Retraction of the supracalvicular fossa^c^	37	16	45	42	47	78
Nasal flaring^c^	41	17	66	62	52	87
Retraction of the suprasternal notch^c^	45	19	48	45	44	73
Scalene contraction^c^	54	23	60	56	52	87
**Non-respiratory physical signs**						
Diaphoresis^c^	5	2	16	15	36	60
Cyanosis ^c^	0	0	0	0	4	7
Alert^e^	182	77	70	65	36	60
Calm^c^^,^ ^d^	212	90	80	75	21	35

Statistical test performed by a non-parametric test for trend across the categories of respiratory distress.

^a^ P <.05,

^b^ P <.01,

^c^ P <.001,

^d^ Calm dichotomizes calm vs. (restless, agitated, or very agitated),

^e^ Alert dichotomized alert vs. (somnolent, stuperous, and comatose).

The least common sign was pursed lip breathing, which was uncommon across all levels of respiratory distress. The most common sign was scalene contraction, which was observed in almost one of four patients who had no or mild respiratory distress. In bivariate analysis, all of the respiratory signs were significantly associated with respiratory distress; however, only Hoover’s sign did not show a consistent increase across the three levels of respiratory distress (Table 4). Cyanosis perfectly predicted severe respiratory distress but was rare.

Diaphoresis was strongly associated with the level of respiratory distress; in particular, there was a four-fold increase in its prevalence from moderate to severe respiratory distress (Table 4). The level of agitation correlated with the level of respiratory distress (Spearman’s rho = 0.39; p<0.001). The percentage of calm patients decreased from 90% in patients with no respiratory distress to 35% in patients with severe distress (Table 4). The level of consciousness correlated weakly with respiratory distress (Spearman’s rho = 0.16; p = 0.01). The percentage of alert patients decreased from 77% in patients in no respiratory distress to 60% in patients with severe respiratory distress (Table 4).

### Ordinal models and bootstrap validation

In the physiologic model (vital signs and presence or absence of hypoxia), RR and hypoxia were independent determinants of the category of respiratory distress; the R-squared for this model was 0.43. When we added readily observed physical signs to the physiologic model (i.e., the limited model), gasping, diaphoresis, and contraction of the sternomastoid muscle remained in the final model and substantially improved the r-squared value (Table 4). When we added the hidden signs for the full model, paradox was the only sign that remained; however, the increase in the r-squared value was negligible. When we evaluated the models’ internal validity using bootstrap methods, the r-squared value remained similar (Table 5).

**Table 5:** Three model with physiologic signs and physical findings most strongly associated with respiratory distress.

	Models						
	Physiologic		Limited			Full	
					bootstrap		
	OR	95% CI	OR	95% CI	OR	OR	95% CI
**Physiologic variables**							
Respiratory rate							
≤ 24	ref	—	ref	—	ref	ref	—
25 to 29	2.4	1.3 to 2.6	2.5	1.2 to 5.4	2.8	2.2	1.0 to 4.8
30 to 34	5.1	2.7 to 9.8	4.3	2.1 to 8.9	4.9	3.9	1.9 to 8.0
35 to 39	18.1	8.8 to 37.2	19.4	8.1 to 46.8	23.8	15.6	6.2 to 39.0
≥ 40	54.3	26.0 to 113	39.8	14.4 to 110	53.4	33.7	12.5 to 91
Hypoxia^a^	4.6	1.4 to 14.7	4.0	1.4 to 11.6	5.2	3.6	1.2 to 11.0
**Physical signs**							
Gasping	—	—	8.8	4.3 to 18.1	10.5	8.6	4.1 to 17.9
Diaphoresis	—	—	5.4	2.5 to 11.4	5.4	4.4	2.0 to 9.7
Sternomastoid contraction	—	—	5.7	3.1 to 10.6	6.4	5.2	2.8 to 9.9
Paradox^b^	—	—	—	—	—	2.2	1.3 to 3.7
**Measures of model fit**							
R-squared	0.43		0.69		0.70	0.70	
Bayesian information criteria	-150		-282			-283	

^a^ Combination of FIO_2_ >40% and oxygen saturation <93%. The models were adjusted for the level of physician’s experience (board certified versus fellow), and for the level of respiratory support (supplemental oxygen alone, on non-invasive ventilation, or during weaning from mechanical ventilation).

^b^ Thoracoabdominal asynchrony or paradox.

## Discussion

We identified the physiologic and physical signs most strongly associated with a physician’s gestalt rating of a patient’s work of breathing. Respiratory rate and hypoxia accounted for a substantial proportion of the variance. By adding diaphoresis, gasping, and accessory muscle use, the model explained the majority of the variance. The five signs can be abbreviated by the mnemonic DiapHRaGM, which represents *diap*horesis, *h*ypoxia, respiratory *ra*te, *g*asping, and accessory *m*uscle contraction.

Because the direct measurement of the work of breathing in a large number of acutely ill, spontaneously breathing patients is difficult and probably unsafe, we had to substitute for it by a strongly associated variable such as dyspnea (symptom) or respiratory distress (physician observation).[8,15,16] Although both are manifestations of increased work of breathing,[17,18] we selected distress because physicians rely on their observations of patients in determining the need for respiratory support and do so independent of patients’ ability to express dyspnea or its severity.[2,19] This choice was reasonable also because the rating of respiratory distress by physicians predicts the need for mechanical ventilation,[4] and because it is as reproducible as dyspnea.[8,20]

The earliest modern account of the signs of respiratory distress was by Gilston who in 1976 described eight facial signs seen in patients with respiratory failure.[21] Patrick and colleagues formulated, based on experience, a scale to describe the increased work of breathing at the moment of instituting mechanical ventilation. It combined the palpated tonic and phasic activity of neck muscles with the presence of retractions and paradox.[22] More recently, Campbell and colleagues validated the 7-item Respiratory Distress Observation Scale (RODS) that was intended to measure respiratory distress in patients under palliative care that are unable to report dyspnea.[23] It included fear, grunting, respiratory rate, heart rate, nasal flaring, accessory muscle use, and restlessness. This scale was accurate in predicting when a nurse would rate respiratory distress as moderate and severe distress.[16] The difference in variables that we identified and those in the RODS is probably due to differences in patient characteristics, study design, and intended application.

Although our physicians were blinded to the vital signs, their quick rating of respiratory distress correlated strongly with respiratory rate, heart rate, and hypoxia. Respiratory rate is the strongest predictor of clinical deterioration of patients on wards and as expected, it was an important variable in the model.[24] Our categories for respiratory rate are supported by existing research. The lowest cut point (24 breaths/min) is the same as the threshold for points in the Acute Physiologic and Chronic Health Evaluation III.[25] A respiratory rate> 30 breaths/min is associated with poor outcome in patients with community acquired pneumonia, and a respiratory rate> 38 breaths/min predicts weaning failure.[26,27] In addition, many of our patients with moderate to severe distress probably meet the definitions of respiratory failure of recent randomized trials because they had similar respiratory rates to patients recruited in these trials and many of them had at least one sign of increased breathing effort.[6]

Hypoxia is not a sign of increased breathing effort but an indicator of the severity of respiratory dysfunction. Nevertheless, it correlated with rating of respiratory distress by a blinded physician. Our a priori definition of hypoxia yields an SpO_2_:FIO_2_ ratio of 200 which gives patients with acute bilateral infiltrates the diagnosis moderate acute respiratory distress syndrome and is usually associated with a moderate shunt.[28,29]

Diaphoresis can be due to effort, hypoglycemia, ischemia, or fever.[30–32] But in patients with acute respiratory illness, it is probably a manifestation of hypercapnia. In patients with acute asthma exacerbation, diaphoresis is associated with a higher carbon dioxide level,[33] and inhaling carbon dioxide increases sweating in normal subjects.[34]

A HR >140 beats/min and an increase by 20% are considered signs of distress in patients weaning from mechanical ventilation.[35] In our study, 10 patients had a HR >140 beats/min and the average HR of patients in severe distress was only 112±22 beats/min—only 8% higher than HR of patients in moderate distress. Nevertheless, we observed that HR increased as the level of distress increased, but it did not improve performance of the physiologic model. An increase in blood pressure is also considered a sign of distress during weaning.[36,37] We found no correlation between it and distress.

Sternomastoid contraction was strongly associated with the level of respiratory distress. At rest, it is inactive in normal subjects and is active only in 10% of patients with severe, stable respiratory disease.[38–41] In contrast, it is frequently active in acute respiratory failure and its activity diminishes with mechanical ventilation,[42] it is recruited in the first minutes of a weaning trial destined to fail,[43] it is active in acute bronchial asthma especially at higher airway resistance,[44] and it is even activated by bronchospasm induced by histamine.[45]

Gasping (switch to oronasal breathing) is normal during exercise at high minute ventilation.[46] Although we did not measure minute ventilation in our patients, the respiratory rate was markedly elevated (33±9 breaths/min) among patients who were gasping; much higher than the respiratory rate when gasping begins during exercise (20–24 breaths/min).[46]

The only other sign of increased breathing effort that remained in the model was paradox. We excluded it because it required the additional examination of the chest and abdomen without improving the model. Others might keep it because it indicates an intolerable work of breathing.[47] The mnemonic becomes DiaPHRaGM with P denoting *p*aradox.

There are three main limitations to our study. First, rating of distress could have biased the ensuing assessment of the physical signs. When we evaluated this concern in a nested analysis of paired, simultaneous observations from the same population, we found that the association between respiratory distress and the signs of breathing effort was similar when the same observer assessed both to when two blinded observers did so.[8] This finding suggested that observer bias had a negligible impact on the observed associations.

Second, if a physician’s rating of distress is valid and reliable, why substitute it with a battery of signs? Relying on a single sign increases the likelihood of measurement error; relying on multiple signs smooths the error caused by the individual signs.[48] But, measuring too many signs (seventeen in our case) is inefficient. To minimize this inefficiency, we reduced the seventeen signs to three plus respiratory rate and hypoxia. Respiratory distress is also more abstract and subjective and hence more difficult to standardize than gasping, diaphoresis, and sternomastoid contraction.

Third, the reliability of the signs of increased breathing effort is suboptimal. This does not preclude their use because the observed high odds ratios have been already attenuated by the signs’ reliability. Improving reliability by training and standardization might improve the model.

Our study has several strengths. Our sample size is larger than that of studies where the work of breathing is directly measured. All of our signs have face validity because they are consistent with physiological principles and standard clinical practice. The final model explains much of the variability in the rating of respiratory distress. The findings are widely applicable because our patients had diseases that affected most organs and not just the lungs. Lastly, the signs are easy to teach and can be easily recalled at the bedside by the mnemonic DiapHRaGM.

In conclusion, a set of five physical signs captures the level of respiratory distress. It may provide a method for rapid, systematic assessment of the work of breathing in acutely ill patients. It also has the potential for improving the examination of patients with an acute respiratory illnesses, enhancing communication between physicians regarding its severity, and standardizing the assessment of research participants with respiratory failure.

## Supporting information

S1 Table Method for performing the physical examination. This table summarizes the instructions given to the physicians during the training on how to examine patients for this study.
(DOCX)

S1 Data Raw physical examination data. (XLS)
