# Supplementary material for: DiapHRaGM: A mnemonic to describe the work of breathing in patients with respiratory failure
Source: PLoS One. 2017 Jul 3;12(7):e0179641. doi: 10.1371/journal.pone.0179641 (PMC5495207; doi:10.1371/journal.pone.0179641)
Supplement: S1 Table — This table summarizes the instructions given to the physicians during the training on how to examine patients for this study. (DOCX) [file pone.0179641.s001.docx]

**S1 Table: Method for Performing the Physical Examination.** This table summarizes the instructions given to the physicians during the training on how to examine patients for this study.

| **Sign** | **Description** |
| --- | --- |
| Body posture | Sitting leaning forward and sometimes resting the forearms on the thighs or on a table or keeping the head lifted above the pillow. |
| Audible breathing | Sounds such as moaning, grunting, wheezing, or stridor heard during inspiration or expiration. |
| Diaphoresis | Sweat observed on the forehead of face |
| Nasal flaring | Outward movement of the nares during inspiration. |
| Cyanosis | Blue or bluish gray discoloration of nail beds, skin, or mucus membranes. |
| Gasping | Breathing with the mouth open and opening the mouth further during inspiration. |
| Pursed lip breathing | Partial closing of lips to slow and prolong expiration. |
| Scalene contraction | Place the tip of two fingers (forefinger and middle finger) just dorsal to the insertion of the sternomastoid on the clavicle into the floor of the posterior triangle of the neck. The sign is present if tension in the muscles felt during inspiration (as a knock, snap or pulsation). |
| Sternomastoid contraction | Drawing the muscle back with the thumb and first finger to feel its contraction. This sign is also present if visible contraction of the sternocleidomastoid muscle is associated with the lifting of the head during inspiration. |
| Tracheal tug | Anchor your pinky finger on the sternum, rest the tip of the index finger on the thyroid cartilage, and feel the thyroid cartilage move downwards toward the diaphragm during inspiration. The sign is present if the thyroid cartilage descends by one finger or more. |
| Retraction of the supraclavicular fossa | Supraclavicular fossa is drawn inward during inspiration. |
| Retraction of the suprasternal notch | Suprasternal notch is drawn inward during inspiration. |
| Hoover’s sign | Rest first and second fingers on the costal margins near the anterior axillary line. The sign is present if the margin is felt to move inwards during any part of inspiration. |
| Thoracoabdominal paradox or asynchrony | Rest one hand on chest and one on abdomen and observe how they move relative to each other during breathing. Asynchrony is present if the chest and abdomen expand at a different velocity and paradox is present if the chest and abdomen move in opposite direction to each other. |
| Abdominal muscle contraction | Rest hands between the lateral sheath of the rectus abdominus and the midaxillary line, and observe the movement of the rectus abdominus. The contraction is present if you feel it especially if you notice inward sculpting of the lateral muscles or anterior bulging of the rectus. |
| Agitation | *Calm:* able to focus, rest, or sleep. *Restless:* anxious but movements are not aggressive. *Agitated:* frequent non-purposeful movements such as shaking head, eyes wide open, gagging. *Very agitated:* pulling tubes, uncontrolled behavior, seeks to get out of bed, violent, unable to rest. |
| Consciousness | *Alert:* fully aware of the environment. *Lethargic*: drowsy but easily arousal, unaware of some elements of environment, not spontaneously interacting with examiner. *Stupor*: difficult to arouse and requires vigorous or repeated stimuli. *Comatose*: not arousal or aware of environment. |
